# Cultural Adaptation of an Aboriginal and Torres Strait Islanders Maternal and Child mHealth Intervention: Protocol for a Co-Design and Adaptation Research Study

**DOI:** 10.2196/53748

**Published:** 2025-01-10

**Authors:** Sana Ishaque, Ola Ela, Chris Rissel, Karla Canuto, Kerry Hall, Niranjan Bidargaddi, Annette Briley, Claire T Roberts, Sarah Jane Perkes, Anna Dowling, Billie Bonevski

**Affiliations:** 1 College of Medicine and Public Health Flinders University Bedford Park Australia; 2 First Peoples Health Unit Griffith University Brisbane Australia; 3 College of Nursing and Health Sciences, Flinders University Bedford Park Australia; 4 The University of Newcatle Newcastle Australia

**Keywords:** Aboriginal, co-design, mHealth, maternal, child health, digital health, children, women, female, cultural adaptation, Torres Strait, research study, Indigenous, digital health intervention, diversity, South Australia, pregnant, health professional, adaption, focus group, pretesting, usability, app, health disparities, information, technology, mobile phone

## Abstract

**Background:**

There is limited evidence of high-quality, accessible, culturally safe, and effective digital health interventions for Indigenous mothers and babies. Like any other intervention, the feasibility and efficacy of digital health interventions depend on how well they are co-designed with Indigenous communities and their adaptability to intracultural diversity.

**Objective:**

This study aims to adapt an existing co-designed mobile health (mHealth) intervention app with health professionals and Aboriginal and/or Torres Strait Islander mothers living in South Australia.

**Methods:**

Potential participants include Aboriginal and/or Torres Strait Islander pregnant women and mothers of children aged 0-5 years, non-Aboriginal and/or Torres Strait Islander women who are mothers of Aboriginal and/or Torres Strait Islander babies, and health professionals who predominantly care for Aboriginal and/or Torres Strait Islander mothers and babies. Participants will be recruited from multiple Aboriginal and/or Torres Strait Islander–specific health services under the local health networks around metropolitan South Australia. In this study, data collection will be carried out via culturally safe, and family-friendly yarning circles, facilitated by Aboriginal research staff to collect feedback on the existing mHealth app from approximately 20 women and 10 health professionals, with the aim to achieve data saturation. This will inform the changes required to the mHealth app. All focus groups and interviews will be audio recorded and transcribed verbatim. Data will be inductively analyzed using realist epistemology via NVivo software (Lumivero). Themes about the mHealth app’s cultural acceptability, usability, and appropriateness will be used to inform the changes applied to the app.

**Results:**

With the feedback received from participating women and health professionals, changes in the smartphone app will be made to ensure the intervention is supportive and meets the needs of Aboriginal and/or Torres Strait Islander mothers and families in South Australia. Participation of community members will promote ownership, community engagement, and implementation.

**Conclusions:**

A co-designed, culturally sensitive, and effective digital health intervention is likely to support Indigenous mothers and their children facing health disparities due to the disruption of Indigenous culture by colaying a foundation for a potential clinical trial and wider implementation.

**International Registered Report Identifier (IRRID):**

PRR1-10.2196/53748

## Introduction

The impact of colonization on Indigenous populations’ health across the globe is well recognized [1]. The ongoing disadvantages of intergenerational trauma and structural violence have resulted in disparities in health outcomes between the Australian general population and Aboriginal and/or Torres Strait Islander communities [1]. Decolonization of interventions, health care organizations, health service provision, and policies are recommended as solutions.

Mothers and babies getting optimal care and support for a good start to life is a priority of the Australian National Aboriginal and/or Torres Strait Islander Health Plan 2013-2023 [2]. However, despite policies, strategies, and funding to mitigate the disparities in pregnancy and birthing outcomes, closing the gap is yet to be achieved [3]. The importance of traditional family and kinship ties and knowledge of birthing and parenting practices remain largely unrecognized in the mainstream health services accessed by Aboriginal and/or Torres Strait Islander communities [4,5]. This contributes to poorer health outcomes experienced by Aboriginal and/or Torres Strait Islander pregnant women, babies, and young children compared with Caucasian Australians [2,4,6]. Aboriginal and/or Torres Strait Islander women of childbearing age (15 years and older) are overrepresented in behavioral risk factors impacting health outcomes. In a recent Australian National Health Survey, a large majority of First Nations women failed to meet guidelines for physical activity, and vegetable or fruit intake [7]. Furthermore, 36% reported daily tobacco smoking, and 35% reported experiencing high or very high levels of psychological distress [7]. The cumulative effects of disadvantage and poorer health outcomes of women subsequently impact infant mortality for Aboriginal and/or Torres Strait Islander babies, which is 2.1 times the rate of non-Indigenous infants [8]. This highlights the urgency of more effective, ground-up, community-led resource and service development [9].

Mobile health (mHealth) apps are being used increasingly for health promotion because of their potential for greater reach [10,11]. Despite their growing popularity, very few evidence-based mHealth interventions for Aboriginal and/or Torres Strait Islander Australians exist [12]. A recent Aboriginal and/or Torres Strait Islanders survey of 398 women aged 16-49 years found that most respondents owned a smartphone, had internet access, and used social media daily [13]. This suggests that a smartphone app may be an effective health promotion tool for Aboriginal and/or Torres Strait Islander women or carers with children.

Evidence-based literature offers many perspectives on co-design. The term “co-design” is often used in research to describe a methodology that involves stakeholders in the “design of services, strategies, environments, policies or processes that impact them” [14]. Digital health solutions designed and developed in the context of mainstream health systems predominantly neglect socioeconomic determinants that are fundamental in understanding and addressing the distinct health challenges that Indigenous communities face [15]. Such an oversight might be rectified by integrating culturally safe co-design practices during the design and development phases [15]. Consequently, this project proposes that the culturally sensitive adaptation of a co-designed mHealth intervention has the potential to significantly benefit the health of many Aboriginal and/or Torres Strait Islander families within South Australia and nationally.

The mHealth program, Jarjums*,* co-designed with Aboriginal and/or Torres Strait Islander people was developed in New South Wales in collaboration with Aboriginal communities and found to be highly acceptable by women and health services [12]. The co-design of the app ensured culture was weaved throughout the project and the mHealth app [12]. The purpose of this mHealth app is to provide information and health behavior change strategies in the domains identified or selected by the co-design participants. The app explores women’s and babies’ health and well-being topics through 6 individual modules. These include (1) smoke-free families, (2) safe drinking, (3) feeling good, (4) women’s business, (5) eating, and (6) exercising. The modules exploring children’s health and well-being include (1) breathing well; (2) sleeping; (3) milestones; (4) feeding and eating; (5) vaccinations and medicines; and (6) ears, eyes, and teeth. Each module includes key messages around the benefit of changing behavior related to illness progression, tips addressing barriers to behavior change, cues for action, and links to further information.

As there are over 250 language or national groups with differing laws and customs across Australia, the current project will develop an adaptation of the intervention for South Australian–based Aboriginal and/or Torres Strait Islander women. The aim of this study is to consult with health professionals, mothers, and carers to culturally adapt a mHealth program called “Growin’ Up Healthy Jarjums” for use by Aboriginal and/or Torres Strait Islander mothers and carers living in South Australia. This research will directly address gaps in the health evidence base and build on existing knowledge by ensuring that programs developed and effective in 1 location, are adapted and tested before they are implemented in the intended population in another location.

## Methods

### Study Design

This is a multisite project and will be conducted at 3 different Aboriginal health care services.

There are 3 levels of involvement of Aboriginal and/or Torres Strait Islander people in this project. These are (1) Aboriginal and/or Torres Strait Islander chief investigators, (2) project governance by a separate Aboriginal and/or Torres Strait Islander Governance Group, and (3) Aboriginal research assistants (ARA).

The Governance Group will oversee the conduct of the project and guide the interpretation of data, dissemination, and translation of study findings. All members have extensive experience working in Aboriginal and/or Torres Strait Islander health care. Specifically, the group comprises research experts, organizational and departmental leaders from various affiliations including universities, government health strategy and research departments, local health networks, maternal and child service providers, and community representatives. The ARAs are local community members and involvement in Aboriginal health services is a crucial component of the research to build trust, safety, and engagement. The ARA involvement also supports capacity building for Aboriginal and/or Torres Strait Islander research.

The framework for the cultural adaptation process will be based on the stages of cultural adaptation stepwise model of Barrera et al [16-19]. Barerra et al [16] describe 5 stages to culturally adapt evidence-based interventions for the purposes of reducing health disparities among culturally diverse populations and include considerations for intercultural diversity. These stages of adaptation include (1) information gathering, (2) preliminary adaptation design (including modifications), (3) preliminary adaptation tests, (4) adaptation refinement, and (5) cultural adaptation trial [16].

In this qualitative study, the existing co-designed mHealth app, Growin’ Up Healthy Jarjums, will be adapted by using focus groups and interviews to collect data on participants’ perceptions, experiences, and ideas about the app. We will conduct sessions using traditional yarning style methodology, to ensure culturally responsive practices are respected. Yarning is an established culturally appropriate process to conduct research with Aboriginal and/or Torres Strait Islander people to both establish rapport and collect information [20]. The sessions will be predominantly conducted by ARAs.

The eligibility criteria include Aboriginal and/or Torres Strait Islander women aged 16 years and older living in South Australia, who are currently pregnant, or mothers of children aged 0 to 5 years. Non-Indigenous women, of similar age are also eligible if they are pregnant with or are mothers of Aboriginal and/or Torres Strait Islander children aged 0 to 5 years. All documents prepared to convey the study information and consent are in English. However, there is a possibility that eligible, interested women may not be able to communicate in English. Such instances will be managed on a case-by-case basis with the support of language interpreters.

Focus groups will be conducted with an estimated 4 to 6 women in each group, up to 20 women in total. It is expected that data saturation will be achieved with 2 to 3 focus groups with approximately 4 to 6 women each. The actual number may vary depending on achieving data saturation.

Recruitment of participants will involve ARAs directly approaching women in the waiting area or room of the participating sites. Information sheets, leaflets, and consent forms will be available to use for recruitment support. Eligible women may directly approach the research team after reading the study information poster at any of the participating sites or the information leaflet provided to them by clinic staff of the participating sites. Furthermore, participating clinics will have project handouts to offer to eligible women.

In addition, participants will be asked if they would like to pass the study information on to a friend or family member who can then choose to connect with the research team to obtain further information and potentially participate. Individuals will be screened for eligibility when they contact the research team by phone. Women who consent to participation will be invited to join a focus group or interview with an estimated duration of 1.5 hours. At the start of the focus group or interview, participants will be asked to complete a face-to-face survey with demographic, cultural, and socioeconomic items. The survey has been adapted from a previously piloted study and deemed to be appropriate by Aboriginal and/or Torres Strait Islander mothers [21,22]. The ARAs will also spread the information to their circle of friends or family and co-workers and recruit via word of mouth.

Health professionals who predominantly care for young Aboriginal and/or Torres Strait Islander mothers or children will also be recruited for interviews. They will be recruited to the study using a separate information sheet and consent form by either the ARAs or the non-Aboriginal and/or Torres Strait Islander Research Fellow involved in the project. This process will involve explaining the participant information sheet and consent form to the health professional. All participation is voluntary, and health professionals will be informed that their decision to participate, or not, will not impact their role or position.

The purpose of including health professionals is to ensure that the messages provided in the app are aligned with the evidence-based messages used within their practice. To garner their views on the use of the app as a tool to connect with Aboriginal and/or Torres Strait Islander families (one of the aims of the app), the appropriateness of the app will also be explored. Health professionals will be interviewed for input into the content and features of the mHealth app until data saturation is achieved.

Once the adaptation process is complete and all the suggested feasible changes are made in the app. Further work regarding the feasibility of the app will be undertaken by asking representative women or carer population to use the app for 4 weeks.

The snowball methodology is predicted to facilitate the recruitment phase of the project. Participating health service managers who agree to support the project will be sent an invitation email to circulate to their staff with the staff study information sheet.

A focus group or interview guide will be used to guide the discussion, although focus groups or interviews will be conducted using an iterative process, with the information gathered from initial focus groups used to guide the dialogue and resources used in sequential groups. Phone interviews may be conducted with participants if this is preferred. If required, an interpreter service will be used to provide support to potential participants who require language interpretation.

The focus group or interview discussion will primarily focus on how an mHealth intervention designed for this community would differ from the “Growin’ Up Health Jarjums” program. Participants will be prompted to discuss images and artwork, language, videos, content, and delivery features of the original app.

All focus groups and interviews will be audio-recorded, with participant consent, and transcribed verbatim by a professional transcription service that will sign a confidential agreement before providing their service.

Participating women and health professionals can withdraw at any time, without giving a reason, throughout the study. Consent to participate can be retracted by contacting the research team; in-person, by phone, or via email. The contact information for the study team is provided on the study information sheets.

If the participant requests a specific response to be removed from the transcriptions, then the researchers will make reasonable attempts to achieve this. However, it may not be possible to remove every detail of a participant’s contributions as it can be difficult to identify individual contributions within the focus group transcriptions.

If participants become distressed during the focus group or interviews when health content for the app is discussed such as child health, smoking or alcohol, and other drugs, a study-specific distress protocol will be adhered to. All events will be recorded and follow-up will be performed.

A generalized data-driven, inductive, thematic analysis will be completed with the transcribed, deidentified data [23]. Inductive analysis is a process in which data coding is undertaken without fitting it into any preexisting coding framework. An ARA and investigator will independently code themes using NVivo software (Lumivero) and complete analysis and write-up with the project team and project Investigators. The data will be interpreted sensitively with the input of ARAs. All personal, identifiable information will remain anonymous in reporting. Deidentified data analysis will be used in manuscripts for publication in peer-reviewed journals and for poster and oral presentations at national and international conferences or symposiums.

The feedback received from women and health professionals will be used to change the app by the technical team. The edited app will be screened by women for acceptability in a follow-up study.

### Ethical Considerations

This project has been granted ethical approval by the Department of Health and Wellbeing Human Research Ethics Committee (2022/HRE00262) and Aboriginal Health Research Ethics Committee (#04-22-1018). A cross-institutional approval has also been obtained from Flinders University. Participation in this study is voluntary. Potential participants will be informed, both in writing on the consent form and verbally during the consent process that they can choose to not participate. Their decision not to participate will not jeopardize their relationship with their organization, health care providers, and research team and will not affect the delivery of care to them. Data will be anonymized during the transcription process and all identifiable, personal details of participants will be removed. Participating women will be reimbursed with a Aus $50 (US $33.64) shopping voucher for their time and provided with refreshments.

## Results

The short-term outcomes of this research will include the start of the development of a culturally appropriate mHealth app to support Aboriginal and/or Torres Strait Islander maternal and child health. It is anticipated that the app will be a tool for women to connect with each other, connect with health professionals, and access evidence-based, culturally responsive health information in a way that is meaningful to them. The design, content, and features of the app will be a collaboration of ideas, stories, and evidence from women, the research team, and health services. Participants will have the opportunity to suggest additional areas of health or additional modules to include in the app. Potential benefits for participating women include opportunities to learn new information, and to engage with and comment on the use of new technologies. Participation of women will also promote ownership and community engagement of the project.

The anticipated long-term outcomes of the research include the contribution of the data to the knowledge on the preferences and potential use of a mHealth app for improving health outcomes of Aboriginal and/or Torres Strait Islander women and children. The resulting data will be used to source further funding opportunities for testing the feasibility, acceptance, and effectiveness of the app in a clinical trial. Ultimately, this app aims to promote health and reduce the rates of noncommunicable and communicable diseases among Aboriginal and/or Torres Strait Islander women and children. Findings may inform local and regional policies and provide advice on how to incorporate these new technologies as part of existing programs and practices.

As of July 2024, the project is ongoing in South Australia. The first phase of data collection will finish in September 2024 and the results will be shared with stakeholders in the form of reports, peer-reviewed publications, and conference presentations.

## Discussion

The data might identify additional needs by participating women and health professionals. That will determine the scope of future research and implementation of mHealth technology related to Aboriginal and/or Torres Strait Islander women and children.

We anticipate that this new digital health intervention will help to reduce health disparities among Aboriginal and/or Torres Strait Islander mothers and their children, laying a foundation for a potential clinical trial and wider implementation.

A strength of this study includes its sensitivity to culturally responsive research methodology. This is woven throughout the study design from the governance model and the inclusion of ARAs to the way in which members of the community will be recruited and the flexibility of the consultation process.

Some anticipated limitations include challenges with recruitment due to the nature of engaging with a specific cultural group. The study does rely on the shared understanding of ARAs with the community and it is anticipated this will foster the development of trusting relationships with participants and support recruitment.

Finally, while the app is currently designed for the South Australian–based Aboriginal and/or Torres Strait Islander community, there is diversity within the community and therefore, this could limit engagement. Questions about how to improve the cultural responsiveness of the app will be asked while gathering feedback.

Concurrently, a recent review on barriers and facilitators of engagement of Indigenous peoples with mHealth interventions revealed themes that echo the need for effective and timely co-design practices in this field [24]. Co-design has been identified as one of the most important factors determining the uptake of web-based therapeutic interventions for Indigenous communities or populations [25]. The review findings support the inclusion of culturally sensitive methodology as well as Indigenous governance and community consultation.

Project outcomes will be provided to organizations and participants while ensuring participants’ confidentiality. Ethical reports and funding body reports will be completed and provided to the relevant figures. We anticipate that there will be numerous opportunities to share and use the data gathered from this research, including dissemination to research institutes, health organizations, and community groups.

Furthermore, this research can inform policy makers and peak organizations of the needs of mothers and families with young children.

## Abbreviations

ARA: Aboriginal research assistant
mHealth: mobile health

## Data Availability

The data that will come out from the study will belong to the Aboriginal and/or Torres Strait Islander Community. Due to the sensitivity required in sharing and spread of that data, it will only be available upon reasonable request made to the corresponding author after approval from the project governance. The corresponding author will review any requests received with guidance from the Aboriginal and/or Torres Strait Islander Governance Group of the project.
